# Tobacco Use and Risk Factors for Hypertensive Individuals in Kenya

**DOI:** 10.3390/healthcare9050591

**Published:** 2021-05-17

**Authors:** Silvia Nanjala Walekhwa, Adnan Kisa

**Affiliations:** 1 Center for Eye Research, Department of Ophthalmology, Institute of Clinical Medicine, University of Oslo, 0450 Oslo, Norway; swalekhwa@gmail.com; 2 School of Health Sciences, Kristiania University College, 0152 Oslo, Norway

**Keywords:** tobacco use, hypertension, Kenya, non-communicable diseases, prevention, health promotion

## Abstract

This study aimed to examine the association between hypertension and tobacco use as well as other known hypertensive risk factors (BMI, waist–hip ratio, alcohol consumption, physical activity, and socio-economic factors among adults) in Kenya. The study utilized the 2015 Kenya STEPs survey (adults aged 18–69) and investigated the association between tobacco use and hypertension. Descriptive statistics, correlation, frequencies, and regression (linear and logistic) analyses were used to execute the statistical analysis. The study results indicate a high prevalence of hypertension in association with certain risk factors—body mass index (BMI), alcohol, waist–hip ratio (WHR), and tobacco use—that were higher in males than females among the hypertensive group. Moreover, the findings noted an exceptionally low awareness level of hypertension in the general population. BMI, age, WHR, and alcohol use were prevalent risks of all three outcomes: hypertension, systolic blood pressure, and diastolic blood pressure. Healthcare authorities and policymakers can employ these findings to lower the burden of hypertension by developing health promotion and intervention policies.

## 1. Introduction

Cardiovascular disease (CVD) is a major contributor to the growing public health epidemic in non-communicable diseases (NCDs) [1]. CVDs account for the most NCD deaths (56.5 million), equating to 18.5 million people annually, followed by cancers (10.0 million), respiratory diseases (4.0 million), and diabetes (2.9 million) [1]. The majority of deaths occur prematurely at an age below 70 years [2]. Alarmingly, most of these deaths are in low- and middle-income countries (LMIC) [3]. CVDs could be referred to as unique conditions of the heart and blood vessels that are essentially and generally caused by common exposure to a trait of a person’s character, defined as a risk factor [1,3]. Among these sets of risk factors are those attributed to habitual behaviors, such as smoking, and metabolic factors, such as hypertension. Furthermore, these risk factors can also be linked to each other, particularly smoking as a risk factor for hypertension [3].

Hypertension is considered one of the dominant risk factors of CVDs, with approximately 1.13 billion individuals exposed to this disease globally. Studies show that hypertension remains undiagnosed in many individuals, especially in sub-Saharan Africa, due to the disease’s hidden symptoms [4,5,6,7,8,9]. In later life, this condition can directly or indirectly lead to premature deaths owing to the complexities that develop in untreated patients or as a result of late diagnosis [4,10,11]. The most common health impacts resulting from high blood pressure include acute myocardial infarction, strokes, cardiac failures, and renal failure, among others [12,13,14,15].

Research findings indicate an escalating rate of high blood pressure mortalities in LMICs, a rate that is equivalent to approximately twice that of high-income countries. For instance, 7 and 25% of individuals in high-income countries and Africa, respectively, are likely to die before their 60th birthday due to high blood pressure [11,16,17,18,19,20]. A systematic review on sub-Saharan Africa also indicated similar findings with varying levels of evidence on the prevalence of high blood pressure in the region, generating a median of 29% [19]. Nevertheless, continuous evidence obtained from various regions indicates a high prevalence of hypertension (between 9 and 50%) among urban settlers and in association with rural–urban migration. Moreover, these findings have been reflected in certain Kenyan studies [21,22,23].

Smoking has been described as a major global risk factor for various CVD conditions, including stroke, peripheral vascular disease, coronary heart disease, hypertension, and cancers, among others [24,25]. Studies have reported that from 63 to 68% of NCDs attributed to death are highly associated with tobacco [26,27]. Nevertheless, a number of deaths occur below the age of 60, with a higher percentage in developing countries than the percentage of deaths in higher-income countries of approximately 29 and 13%, respectively. Furthermore, an estimated 12.7% of the 7 million tobacco deaths are due to secondary smoke exposure [28].

Smoking and hypertension individually pose alarming consequences, and this concern is heightened when they are both associated with the same individual [29,30]. This particular modifiable risk needs to be better understood, allowing the development of suitable control measures, which may result in improved health and reduced mortality and morbidity due to the elimination of the double shared risk it poses. Information on ill health within sub-Saharan Africa is scarce but is not different from the situation found in Kenya [31]. To our knowledge, no study has examined the relationship between smoking and hypertension indices in Kenya using a large, nationally representative, and population-based data set. Accordingly, this study examines the association between tobacco use and hypertension in the Kenyan population. The study further controls for BMI, waist–hip ratio, alcohol consumption, physical activity, and socio-demographic or economic factors to establish the prevalent link to hypertension among the Kenyan population. The research questions posed are: how is tobacco use associated with hypertension, and what are the risk factors in hypertensive individuals in Kenya?

## 2. Materials and Methods

### 2.1. Study Population

To understand the association between tobacco use and hypertension, the study utilized the 2015 Kenya STEPs survey provided by the Kenya National Bureau of Statistics (KNBS) and Kenya’s Ministry of Health (KMOH) in conjunction with the World Health Organization’s (WHO) global approach, which is a STEPwise surveillance strategy of NCD risk factors [32,33]. The data set represents the details of an all-inclusive national survey, which is the first one to ever be done on NCD risk factors and injuries. Moreover, the systematic design of the surveillance strategy employed was set to provide a platform of comparison within and across countries [34,35]. Ethical permission was obtained from the KMOH for the use of the data.

The 2015 Kenya STEPs survey was a national cross-sectional household survey designed to provide estimates for indicators on risk factors for NCDs for people aged from 18 to 69 years [35]. The sample was designed with a sample size of 6000 individuals to allow national estimates by gender and residence. The 5th National Sample Surveys and Evaluation Programme (NASSEP V) master sample frame, which was based on the 2009 Kenya Population and Housing Census clusters, was used for the survey [32].

A three-stage cluster sample design was employed for the survey. In the first stage, 100 urban and 100 rural clusters were selected from one sub-sample of the NASSEP V frame. A uniform sample of 30 households from the listed households in each cluster was also selected in the second stage of sampling. The final stage of sampling was completed using the personal digital assistants (PDAs) at the time of the survey and involved the selection of one person randomly from all eligible listed household members using a programmed KISH method of sampling [32,33,34].

To answer the research questions posed in this study and to further understand the impact of tobacco use associated with hypertension, pregnant women were excluded from the study sample. Pregnancy is known to induce hypertension, which is referred to as gestational hypertension among the normotensive or chronic hypertension among females who are hypertensive before pregnancy or within the first 20 gestational weeks [36]. Consequently, pregnant females were excluded because there were no variable measures in the data that could control for these observations in the model [36]. Of the study population from the household sample of 6000, only 92% of those occupying the households consented to the survey, which reduced the number of individuals to a final 4500 individuals who consented to a full interview. Of this group and for this study, 3% of the subjects were pregnant, 0.3% constituted outlier values, and analysis included only those who had less than 5% of the missing values, thereby resulting in the exclusion of 0.02% observations and retaining 4272 subjects for analysis.

### 2.2. Data Collection Procedures

Data were collected in up to three steps using a structured tool developed by WHO STEPwise between 9 April 2015 and 10 June 2015. The data collection tool was developed by 20 trained teams of research assistants and health workers. The initial stage uses interview-based questionnaires to collect information on socio-demographic and behavioral risk factors as well as the individual’s history of diabetes and hypertension. The second stage involves the physical measurements of weight, height, waist circumference, blood pressure (BP), and pulse rate. The final stage involves the collation of biochemical measurements of fasting blood glucose and cholesterol [32,34].

### 2.3. Study Measures

#### 2.3.1. Dependent Variables

Three outcome variables were used in this study: hypertension, systolic blood pressure (SBP), and diastolic blood pressure (DBP). However, the core dependent variable in this study was hypertension. Any participants were considered hypertensive if they were found to have an SBP higher than or equal to 140 mmHg and/or a DBP higher than or equal to 90 mmHg or reported current antihypertensive therapy use [37,38]. The hypertension variable is classified in this study as a binary outcome on the averages of the three readings of blood pressure.

Nadar and Lip [30] proposed that the focus of the risk assessment should be on SBP to necessitate therapy. Nevertheless, tobacco use may influence these two variables differently, and the elevation and reduction of systolic or diastolic pressure (DP) help to explain the level of hypertension. Thus, these two variables were considered continuous and estimated on the arithmetic mean of the three measurement readings performed during the survey. These measurements were performed on the same day with a 2–5 min variance with the help of clinical personnel using (OMRON^®^, Kyoto, Japan), an automated blood-pressure measuring instrument [34].

#### 2.3.2. Independent Variables

The independent variables included demographics (age, sex, marital status, place of residence, occupation, education level, and wealth), personal and family history of hypertension, behavioral measures (current consumption of smoked and non-smoked tobacco products, intensity of physical activity), and physical measures (weight, height).2.4. Reliability and Validity

First, the survey provided comprehensive information on risk factors to the subject and robustness in sample selection (KMOH and WHO). Moreover, the survey’s high response rate allows for the generalization of this study to the relevant population in Kenya. Second, the statistical methods applied in the study have been tested by various studies, and they were used in determining the risk factors associated with hypertension in different settings. In addition, the variables and the measures that were employed in this study are recommended by international bodies and have also been tested in other studies. Third, the procedure employed for physical blood-pressure measurement is considered highly robust in ascertaining the true blood pressure of the subjects [34]. However, as Pickering et al. [37] suggested, not all subjects were measured in the mornings or evenings as the process undertaken was a continuous one and took place throughout the day as individuals were measured immediately after their interviews.

### 2.4. Statistical Analyses

Descriptive statistics, Pearson correlation, frequencies, and regression analyses (linear and logistic) were used to execute the statistical analysis in IBM SPSS 25 and Stata 15 (IBM, Armonk, NY, USA). The variables’ significance was evaluated using chi-square for the categorical variables and the t-test for the continuous variables. All *p* values of less than 0.05 were considered to be statistically significant.

Using a backward stepwise regression, variables for inclusion in the final model were identified through the significance and rating of Akaike’s information criterion and the Bayesian information criterion (AIC&BIC) by selecting the model that had the lowest AIC&BIC. Nonetheless, more weight was attributed to the rating from the BIC for selection. BIC was afforded a higher weighting because its rating methodology is based on the awarding and/or penalizing for variable contribution to the model, while the AIC only penalizes [39].

The developed model was considered to be the one that provided the best fit with a high R2 that represented an explanatory power of the independent and the control variables. The model was then tested on the three outcomes using weighted data. Even though the study sample size was quite large to average in normality, the assumptions for the models were also tested by applying the Kruskal–Wallis tests [40,41].

The models are elaborated below:

- Ysystolic = β0 + β1tobaccouse + β2age + β3gender + β4alcohol + β5BMI + β6marital-status + β7waist-hipratio + β8Physcal-Activity + β9Education + β10wealth+ β11region + β12work
- Ydiastolic = β0 + β1tobaccouse + β2age + β3gender + β4alcohol + β5BMI + β6marital-status+ β7waist-hipratio β8Physical-Activity + β9Education + β10wealth+ β11region + β12work
- Yhypertension = ln (π/1 − π) = β0 + β1tobaccouse + β2age + β3gender + β4alcohol + β5BMI + β6marital-status + β7waist-hipratio+ β8Physical-Activity + β9Education + β10wealth+ β11region + β12work

## 3. Results

This study aimed to investigate the association between tobacco use and the development of hypertension as well as other known hypertensive risk factors in Kenya. The population was characterized as follows:

### 3.1. Characteristics of the Study Population

The study participants included in this study ranged between 18 and 69 years old, with 58.8% of all participants identified as females (2559) after excluding all pregnant women. Moreover, the number of women in all the rural–urban settings was high within each of the wealth quantiles, as shown in Table 1. Each quantile represented approximately 20% of the participants. The participants’ mean age was 37.82 ± 13.46 with almost 70% below 44 years, and about 67% were married, and 40 and 9.9% were self-employed or unemployed, respectively. Homemakers made up 24.5% of participants, and at least 47% had completed their primary education or 7.7 ± 5 years of school. In general, 51% of all participants lived in a rural setting across the country. Among the hypertensives, 36.6% were males, and 51.9% were urban dwellers. The difference within the groups was not significant at the 0.05 level. Within the marital status and age groups, approximately two thirds of the hypertensives were married and between 30 and 59 years of age, while the self-employed and homemakers were also more hypertensive within the occupation status at 40 and 24%, respectively. In the wealth quantile groups, the poorest had the lower percentage (13.5%) among hypertensive, while the middle, fourth, and fifth (richest) quantiles were higher at approximately 22% each.

Higher proportions of the demographic categories among current tobacco users were identified in males (79%), in the age groups between 30–44 (41%) and 45–59 (27%), the married (64%), the self-employed (43%) and employed (21%), and the rural dwellers (56%). In the wealth quantile category, an estimated one fifth were associated with the second and middle quantiles, whereas most of the current tobacco users were the poorest (30.6%). Among the former tobacco users, similar groups as those in the current tobacco users had higher numbers presented as quitters except the wealth quantile groups where the poorest were indicated as having the lowest percentage of quitters at 12.8%.

**Figure 1:**
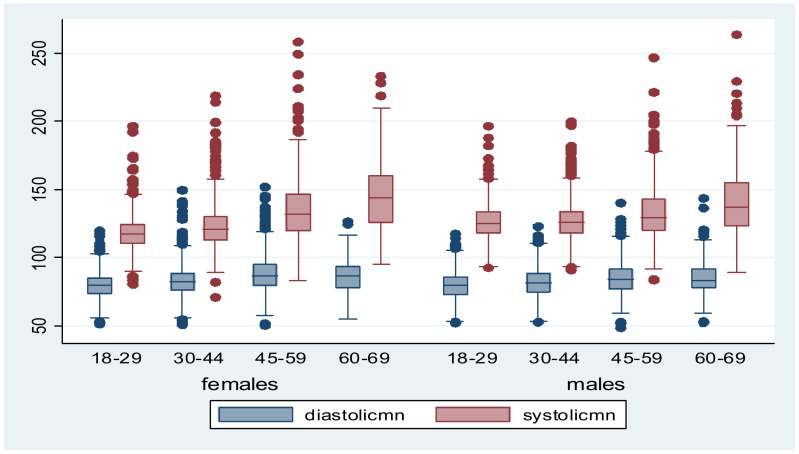
Systolic and diastolic distribution in both and by genders box plot.

Table 2 lists the descriptive statistics of physical measures and continuous variables. The results indicate that the two parameters, systolic and diastolic blood pressure, had exceptionally low and extremely high markers (diastolic 48–152 mm Hg and systolic 71–264 mm Hg) with a mean of 83 ± 12 and 127 ± 20 mm Hg for the diastolic and systolic blood pressure, respectively. These two parameters also varied among males and females and were found to have a greater degree of skewing to the right among two age groups of 30–59, as illustrated in Figure 1. The mean of the waist–hip ratio (WHR) of participants is above the recommended normal ratio for women at 0.8±0.08 while the BMI mean is just below the overweight threshold at 23.5 ± 5.08 kg/m^2^. The fasting blood glucose mean was reported at 4.7 ± 1.4, and the mean of physical activity was noted to be 6 ± 4.2 h per day with at least no hours of physical exercise for others.

### 3.2. Prevalence of Risk Factors among Participants

The prevalence of hypertensive participants was reported as 22%, as noted in Table 3. Moreover, the findings indicated that tobacco consumption and former tobacco users among the participants were 12.6 and 8%, respectively. More people were exposed to alcohol drinking than tobacco use, with a prevalence of 21%. Furthermore, obesity levels were reported as high, based on BMI (obese 10%, overweight 21%) and WHR (obese 25%, overweight 26%). However, the raised blood glucose levels and physical activity levels of less than 30 min were both found to be relatively low compared to all other risk factors, and both were reported as 6%. The highest prevalence was found in fruit and vegetable consumption of less than five servings, which was reported as 95 and 96%, respectively (see Table 3).

### 3.3. Prevalence of Risk Factors among Hypertensive Participants

Among the hypertensive females, 4.3% were current tobacco users, compared to 23.4% of males. Approximately 50% of men were found to consume alcohol compared to only 8.7% of females. Additionally, 41.3% of males and 25.2% of females were exposed to second-hand smoke. Most males had a WHR of <0.9 (59.4%), or they were overweight (34.3%), while most females were obese (53.4%) and overweight (26.3%) when classified according to their WHR. However, when the BMI classification was used, only 25.8% of females were obese, and 33.2% were considered overweight, while 22 and 7% of males were classed as overweight and obese, respectively. Most of the males were found to have a normal BMI (48%). All the differences were found to be statistically significant in all the categories mentioned above except for a few factors. First, the raised blood glucose was found to be high (13.1%) among females compared to males (10.6%). Both females and males were reported to have low vegetable consumption, low fruit consumption, and less than 30 min of physical activity per day.

Table 4 lists the health practices noted in individuals that are also indicated as hypertension risks. These health practices could lead to self-awareness and an understanding of the distribution in healthcare facility utilization. The major primary source of healthcare for most Kenyans is a dispensary or community health worker (34%), followed by referrals to public hospitals (28%), which were previously referred to as district and provincial hospitals. This seems to be the preferred source in almost all categories of the explored risk factors. Specifically, over one third utilized dispensaries in each group, while more than a quarter utilized referrals to public hospitals. There is also a high rate of self-medication or use of alternative therapy (9%), which is also highly preferred among former tobacco users (17.4%), current tobacco users (14.2%), current alcohol users (13.8%), obese individuals (12.2%), and those considered WHR overweight (11%).

Of the participants, almost 51% had never been checked for blood pressure, and within this group, the majority of current tobacco and alcohol users had never been checked (71 and 62%), respectively. In contrast, over two thirds of those classed as obese and overweight, and those with raised blood glucose were more likely to have been checked for hypertension. Among those checked for hypertension, 50% of individuals with raised blood glucose were diagnosed to be hypertensive, while almost 25% consisted of individuals from the other risk factors (BMI, WHR, alcohol consumption, and tobacco use). Over 50% of the diagnoses had occurred during the 12 months prior to the survey. Nevertheless, medication adherence seems to be low, with approximately 60–80.8% not taking the prescribed medication within all the groups, and the highest non-conforming group being the past alcohol users.

### 3.4. Linear Regression of Systolic Blood Pressure and Diastolic Blood Pressure on Tobacco Use

The backward stepwise regression was used to establish a fitting model, and the one presented here was found to have a higher explanatory power (R2) and a low BIC score (40). Consequently, a comparative model was run with all the variables to determine the difference from the main model. The models were run on an unweighted scale first, followed by a weighted model. The unweighted bivariate model indicated non-significant results on current tobacco users and estimated 6.9 mmHg higher SBP in former tobacco users. These measures and statistical significances were altered after controlling for other explanatory variables and are now presented in weighted models below.

Using the weighted model and after controlling for the relevant variables, the association with the development of systolic pressure for current tobacco users was found to be 3.14 mmHg lower than the systolic pressure of never tobacco users. A 1.5 mmHg higher systolic mean had been predicted in the bivariate model, although it was deemed non-significant. On the other hand, former tobacco users’ low mmHg systolic pressure (0.76) means compared to never-tobacco users could not be justified statistically. This was despite the level in the bivariate model being observed to be 7.4 mmHg higher than that of never-tobacco users. In addition to current tobacco users, several other factors were associated with a higher SBP. These included ages 60–59 (22), 45–59 (11.6), 30–44 (1.9), males (5.9), never married (1.7) students (3.0), current alcohol users (4.1), BMI (overweight 5.0 obese 8.8) WHR (overweight and obese approximately 2.4), former alcohol users (3.3), or reduction of SBP: underweight (6.4).

A weighted bivariate and multiple linear regression model was then used to define the association between DBP and tobacco use. However, this model could not statistically confirm the low mmHg measure of DP in current users (0.76) and former users (1.15). Nevertheless, an association was identified between higher mmHg measures and DBP in the categories of age range 60–59 (6.2), 45–59 (6.4), 30–44 (2), current alcohol use (2.7), BMI (overweight 3.2 and obese 6.3), and WHR (overweight 1.7 and obese 1.3).

### 3.5. Multiple Logistic Regression Model

The association between tobacco use and the development of hypertension, as previously mentioned, was determined by logistic regression at a 95% confidence interval (CI). In a weighted bivariate logistic regression model, estimates indicated 1.48 (CI: 1.16–2.3) higher odds of hypertension in former tobacco users, while current tobacco users, even though non-significant, had 0.94 (CI: 0.77–1.44) odds. However, in a multivariate model, tobacco use remained non-significant with less likelihood of 0.12 (0.88–CI: 0.61–1.28) and 0.1 (0.99–0.64–1.53) current and former users, respectively, as observed in the bivariate model. Therefore, significant odds for the risk associated with developing hypertension were found and included age with those who were older found to have a higher risk. Specifically, the highest risk was reported in those aged 60–69 with odds of 5.3 (CI: 3.70–7.46), followed by the 45–59 age group with odds of 3.53 (CI: 2.6–4.80), and the 30–44 age group was found to have odds of 1.33 (CI: 1.00–1.76). Other identified risks included BMI >25 kg/m^2^ with odds of 2.6 (CI: 1.9–3.6), WHR overweight with odds of 1.4 (CI: 1.07–1.7), obese with odds of 1.6 (1.25–2.09), current alcohol users with odds of 1.68 (CI: 1.24–2.3), and underweight BMI, which was associated with lower odds of developing hypertension (0.55) (see Table 5). Thus, in this model, the constant of 0.072 odds (0.04–0.12) was linked to the base factors that included females of 18–29 years of age of normal BMI who never used tobacco or alcohol and were married. Moreover, it was also linked to the base factors that despite being poor, they had finished secondary school, were also employed, and had lived in a rural area with high levels of physical activity.

### 3.6. Sensitivity Analysis

The comparison models on the effects of the explanatory variables had minor differences from the main models. These were noted in the finding of associations between SBP and physical exercise that was less than 30 min a day of 3.8 mmHg lower than the systolic means of those who would undertake a physical activity for more than 60 min per day. Moreover, it was also identified in individuals who ate the recommended quantity of vegetables and had a lower (4.5 mmHg) SBP than those who did not consume five vegetables a day. Furthermore, a unit increase of cholesterol resulted in a 1.2 mmHg increase of systolic pressure, and for tobacco use, this resulted in a 3.7 mmHg lower level in current users, while the student effect was no longer significant as the net of all other variables (see Table 6).

Conversely, DP was associated with a 4.6 mmHg lower diastolic means in those who were unable to work or had retired. Likewise, a unit increase of cholesterol was linked to a 0.9 mmHg increase in diastolic blood pressure, and a raised blood glucose level increased the diastolic means by 2.52 mmHg. Additionally, the indication of current tobacco users changed to signify a higher diastolic mean of 0.25 mmHg which was considered not significant, while the WHR obese effect of 1.3 mmHg was reduced to non-significant.

In the logistic model, a raised blood glucose was linked to higher risks of hypertension with 1.6 odds, while the effect of the 1.3 odds on 30–44 years old was not significant. The coefficient and odds ratio changes ranged between ±0.02 to ±1.5 in all variables. Nevertheless, the comparison models analyzed the effects using a smaller sample (*n* = 3057) than that employed in the main model sample (*n* = 4272). This was due to the exclusion of participants with missing data for some variables, as the analysis used complete case analysis.

## 4. Discussion

In our first research question tobacco use could not statistically justify the protective factor among the current users for all the study outcomes (systolic, diastolic and hypertensin). While former tobacco users were likely to be hypertensive only in a bivariate perspective, which does change by controlling for all the other factors though not statistically significant. Among the other factors in our second research question BMI, current alcohol use, waist–hip ratio, and age were significantly associated with hypertension. We discuss further these estimates and other findings below.

The overall prevalence of hypertension in men and non-expectant women within the age range of 18–69 was 21.7%, which was below the global level and that of LMIC (31.1%) in accordance with the 2010 estimated prevalence (8). The prevalence was also well within the range of the various studies conducted in different settings in Kenya (6.7–50%) and sub-Saharan Africa (6–48%) [4,42,43]. The differences in the prevalence in the various studies could be attributed to the population’s heterogeneity and the variance in behavioral risks within these groups. For instance, a study in Kenya that reported a prevalence of 6.7% was conducted in an urban setting among males over 15 years who were returning from a mosque. In addition to their cut-off point (>140/>90 mmHg), this study undertook a subjective decision in classifying individuals as either hypertensive or not, and this subjectivity could have affected the results [43]. On the other hand, the highest prevalence was attained in a cross-sectional study that was conducted on individuals who were 50 years or older and in an urban setting. Accordingly, such a setting would potentially result in a higher prevalence [5].

Contrary to several studies that show higher prevalence of hypertension in men [41,44,45,46,47,48,49], this study found higher hypertension prevalence in women (23.4%) than men (19.3%), which were similar to South African and Tanzanian studies that found females to have higher prevalence though South Africa prevalence was seen to decrease in women with higher education [47,50]. In addition, as in many other sub-Saharan countries, higher prevalence was among 60–69 years old 42%, the retired or those unable to work 38%, 45–59 year old 35%, the widowed 34%, and divorced or separated 27% [20].

### 4.1. Hypertensive Awareness

Generally, hypertensive awareness in most African regions has been low. As indicated in the survey used in this study, more than 50% had never been tested for their blood pressure. Such a lack of testing makes it even more difficult to control the epidemic if such practices are not reversed. These findings were in contrast to studies conducted in regions of the Americas or Europe [1,3]. For instance, one study in Brazil had indicated full awareness in the entire adult population, with at least one fifth of participants who did not adhere to medication [48]. However, in this study, the participants in the different risk factor groups were reported to have a lower knowledge trend of their hypertensive status. Among current tobacco users and alcohol users, an estimated two thirds were not aware of their status or had never been tested for hypertension. In addition, those diagnosed within this group were less likely to follow the prescribed treatment, with approximately seven in every ten failing to meet treatment requirements.

Furthermore, the findings indicated that most health workers were more likely to check hypertension in individuals who were either overweight or obese or tested for impaired glucose or had a raised blood glucose level compared to other risk factors. This could be due to the apparent knowledge of the blood pressure risk among these groups, even though the participant’s adherence to treatment remains below one third except the raised blood glucose group.

### 4.2. Hypertension Risk Factors

The prevalence of tobacco use was reported as 12.6% and does not differ greatly from the national survey findings [32]. However, the results indicate that there was a higher prevalence of tobacco use in males (23%) compared to females (4%) among the hypertensive. Nevertheless, hypertensive females were also characterized as obese or overweight or with a BMI greater than 25 kg/m2 and were more likely to be exposed to second-hand smoke. Tobacco use was low in this group, but alcohol use was substantially higher (9%). Conversely, 50% of hypertensive males consumed alcohol, while 40% were exposed to second-hand smoke. These findings could potentially be explained by the reasoning that those who drink also smoke. Accordingly, those non-smokers who drink are more likely to be exposed to such second-hand smoke [49]. In addition, they were also characterized as overweight, while a few were obese compared to women.

In the study, current tobacco use failed to meet researchers′ expectations of increasing SBP. Instead, current tobacco use was linked to 3.14 mmHg lower systolic means compared to never tobacco users. Furthermore, its effect on DBP could not be confirmed using the study data, and this was similar to the logistic regression results, as lower odds were observed in current tobacco users. The odds of current tobacco users did not differ when analyzed differently as smokers or smokeless users even after controlling for all other variables. Thus, as in previous studies that failed to establish a link of either smoking or smokeless tobacco with hypertension, this study also reported findings that were contrary to the findings in a study performed in Yala Kenya with odds of at least two among current smokers and former smokers. The Yala study was supported by other studies, such as a Rwandan study that found almost 1.5 odds in current smokers as well as former smokers, 2.3 odds in smokeless tobacco use of DP among rural Indians and Bangladeshis, and 3.5 odds for smokers, while tobacco use were only found to be significant in bivariate analysis [8,41,46,49,50,51,52,53]. Nevertheless, Green, Jucha, and Luz (1986) indicated that blood pressure may acutely be escalated due to smoking but shows a lower blood pressure among smokers than never smokers or former smokers [54].

This study indicates the magnitude of the age effect on hypertension and diastolic or SBP. Hypertension was found to be even higher in the older population with odds of 5.3, a 6.2 mmHg higher diastolic means, and 22 mmHg higher systolic pressure in 60–69 year-old. In contrast, among the 45–59 age group, the magnitude was a 6.4 mmHg higher diastolic mean, an 11.6 mmHg systolic means, and hypertension odds of approximately 3.7 [4,44]. Additional risk factors that were associated with all the outcome parameters accessed included BMI, alcohol consumption, and WHR with at least 1.04–2.6 odds of hypertension, a 1.3–6.3 mmHg higher DP means, and a 2.5–8 mmHg higher means of systolic pressure.

However, participants who were male, past alcohol users, and single, and a student were also associated with a SBP with a 1.6–5.9 mmHg higher means while current tobacco use and an underweight BMI were identified as protective factors of SBP. On the other hand, contrary to several studies, living in urban or rural settings was not significantly associated with developing hypertension (systolic or diastolic) blood pressure nor was the level of wealth, education or the level of physical activity, and the marital status (divorced, separated or widowed) [23,41,42,55,56,57,58]. Similar results were also found in Uganda with the exception of alcohol use [59]. It is also worth mentioning that even though the results were not significant, they were consistent with the findings in the contradictory studies above. Hence, the outcomes in this study could have been affected by the size within some groups, which might have affected their statistical power to influence the results. For instance, within marital status, the married (analysis base) sample proportion was 67.2%, while the widowed (1.2 odds) or divorced/separated (1.1 odds) had a proportion of an estimated 7% each.

### 4.3. Strengths and Limitations

The data used in this study are the most recent and provide relevant information on demographics as well as behavioral characteristics of the sample participants from the national population. Furthermore, the sample in the study was proportionally balanced, especially with regard to gender, region, and wealth quantiles. Hence, the results of these variables had enough statistical power to affirm their specific relationship to the study outcomes. Thus, it is probable that the results that were achieved here are the true effect of the outcomes. Some limitations include the limited data on various dietary risk factors, such as salt, which is known to have a detrimental association with hypertension. Moreover, there was a larger proportion of missing data on cholesterol, fasting blood glucose, fruit, and vegetable intake, which led to the exclusion of results used in the main models of analysis. On the other hand, blood pressure measurements were not taken within recommend time hence conditions surrounding blood pressure variation might have affected the findings [37].

### 4.4. Further Research

The findings in this study enrich the body of research into this area and can act as a cornerstone for further research that should consist of standard measures, especially regarding assessing the dietary salt aspect that was not analyzed in this study. Tests on interactions, though beyond the scope of this study, were not able to be predicated. However, the effect of tobacco use, alcohol, and physical exercise interactions could be studied to establish if either of them had an influence on pre-eminent the risk of the other.

## 5. Conclusions

This study reports the association between tobacco use and the burden of hypertension. The findings also illustrate an important burden of hypertension in the Kenyan population. Kenya is in line to combat NCDs and has developed various policies that endeavor to integrate existing national and global initiatives with the ultimate aim of reversing NCDs by 2020. Therefore, the findings of this study and those found in regional or specific settings in Kenya could be used as evidence in the prevention of hypertension and to support the equipping of healthcare facilities for screening those at risk. There is a need for greater awareness of hypertension in the general population and among healthcare givers. In addition, within the health system, greater emphasis needs to be placed on the detection, treatment, and control of high blood pressure. In conclusion, health policies in Kenya must subsequently account for the control of hypertension. Thus, health policies and health promotion programs should be developed at all levels for health promotion and prevention.

## Figures and Tables

**Table 1:** Demographic characteristics of participants’ hypertension status and tobacco use status.

		Overall *n* (%)	Hypertension		Prevalence	Tobacco Use		
			No	Yes		Never	Current	Former
			*n* (%)	*n* (%)		*n* (%)	*n* (%)	*n* (%)
Gender	Females	2559 (58.8)	1959 (57.5)	600 (63.4)	23.4	2379 (68.6)	115 (21.0)	65 (19.0)
	Males	1793 (41.2)	1447 (42.5)	346 (36.6)	19.3	1090 (31.4)	433 (79.0)	270 (81.0)
Age groups	18–29	1406 (32.3)	1245 (36.6)	162 (17.1)	11.5	1259 (36.3)	93 (17.0)	54 (16.1)
	30–44	1660 (38.1)	1359 (39.9)	301 (31.8)	18.1	1313 (37.8)	226 (41.2)	121 (36.1)
	45–59	873 (20.1)	565 (16.6)	308 (32.6)	35.3	627 (18.1)	153 (27.9)	93 (27.8)
	60–69	413 (9.5)	237 (7.0)	175 (18.5)	42.4	270 (7.8)	76 (13.9)	67 (20.0)
Marital status	Single	774 (17.8)	668 (19.6)	106 (11.2)	13.7	648 (18.7)	78 (14.2)	48 (14.3)
	Married	2926 (67.2)	2289 (67.2)	637 (67.0)	21.8	2347 (67.7)	352 (64.2)	227 (67.8)
	Divorced/separated	306 (7.0)	223 (6.6)	83 (8.7)	27.1	474 (13.7)	118 (21.5)	60 (17.9)
	Widower	346 (8.0)	226 (6.6)	120 (12.7)	34.7	265 (7.6)	52 (9.4)	30 (9.0)
Main work	Employed	822 (18.9)	631 (18.5)	191 (20.2)	23.2	618 (17.8)	117 (21.4)	87 (26.0)
	Self-employed	1744 (40.1)	1364 (40.0)	380 (40.2)	21.8	1342 (38.7)	236 (43.1)	166 (49.6)
	Unemployed	433 (9.9)	345 (10.1)	88 (9.3)	20.3	307 (8.8)	85 (15.4)	41 (12.2)
	Homemaker	1066 (24.5)	838 (24.6)	228 (24.1)	21.4	955 (27.5)	86 (15.5)	25 (7.5)
	Student	184 (4.2)	164 (4.8)	20 (2.1)	10.9	174 (5.0)	4 (0.7)	6 (1.8)
	Others (retired or Unable to work)	103 (2.4)	64 (1.9)	39 (4.1)	37.9	73 (2.1)	20 (3.6)	10 (3.0)
Residence	Rural	2233 (51.3)	1778 (52.2)	455 (48.1)	20.4	1759 (50.7)	309 (56.4)	165 (49.3)
	Urban	2119 (48.7)	1628 (47.8)	491 (51.9)	23.2	1710 (49.3)	239 (43.6)	170 (50.7)
Wealth quantile	1 Poorest	867 (19.9)	738 (21.7)	129 (13.6)	14.9	659 (19.0)	165 (30.1)	43 (12.8)
	2 Second	871 (20.0)	687 (20.2)	183 (19.3)	21	675 (19.5)	120 (21.9)	76 (22.7)
	3 Middle	870 (20.0)	657 (19.3)	213 (22.5)	24.5	683 (19.7)	110 (20.0)	77 (23.0)
	4 Fourth	875 (20.1)	662 (19.4)	213 (22.5)	24.3	710 (20.5)	80 (14.6)	85 (25.4)
	5 Richest	869 (20.0)	662 (19.4)	208 (22.0)	23.9	742 (21.4)	73 (13.3)	54 (16.0)

**Table 2:** Descriptive statistics of physical measures and continuous variables.

	Mean	Standard Deviation	Minimum	Maximum
Systolicmn	127.44	19.91	70.67	263.67
Diastolicmn	82.61	12.1	48.33	151.67
Fasting blood glucose	4.68	1.36	1.1	24.3
Waist–hip ratio	0.85	0.08	0.36	1.49
Body Mass Index (BMI)	23.48	5.09	11.4	75.16
Hip circumference	94.06	13.1	45	165
Waist circumference	79.6	13.73	30	155
Weight	62.77	13.36	30	171.3
Height	163.76	9.36	101	194.5
HDL cholesterol	3.69	1.01	2.5	10.3
Age in single years	37.82	13.46	18	69
Years spent in School	7.71	4.98	0	30
Physical Activity (min)	370.34	253.05	0	1830

**Table 3:** Risk factors in the full sample and people with hypertension by gender.

		Females (*n* = 600)			Males (*n* = 346)			χ2 (df)	Overall (*n* = 4352)
		%	Confidence Level 95%		%	Confidence Level 95%			%
Tobacco usage	Never	91.2	88.7	93.2	57.5	52.3	62.6	149.2 * (2)	79.7
	Current user	4.3	2.9	6.2	23.4	19.2	28.1		12.6
	Former	4.5	3.1	6.4	19.1	15.2	23.5		7.7
Waist–hip ratio	Underweight/normal	20.3	17.1	23.7	59.4	54.0	64.7	219.5 * (2)	48.5
	Overweight	26.3	22.8	30.0	34.3	29.2	39.6		26.2
	Obese	53.4	49.3	57.5	6.3	4.0	9.4		25.3
Body mass index (BMI)	Underweight	4.1	2.7	5.9	10.1	7.1	13.7	52.1 * (3)	12.0
	Normal	36.9	33.0	41.0	53.1	47.7	58.6		56.8
	Overweight	33.2	29.4	37.2	26.7	22.1	31.8		20.7
	Obese	25.8	22.3	29.5	10.1	7.1	13.7		10.4
Alcohol consumption	Never	77.0	73.5	80.2	37.0	32.0	42.2	209 * (2)	66.5
	Past	14.4	11.7	17.3	13.9	10.5	17.8		12.4
	Current	8.7	6.6	11.1	49.1	43.9	54.4		21.2
Second-hand smoke	No	74.8	71.2	78.1	58.7	53.4	63.8	26.59 * (1)	68.5
	Yes	25.2	21.9	28.8	41.3	36.2	46.6		31.5
Vegetables consumed per day	Low (<5 servings/day)	96.7	95.0	97.9	98.5	96.6	99.4	2.49 (1)	96.3
	Adequate (>5 servings/day)	3.3	2.1	5.0	1.5	0.6	3.4		3.7
Fruit consumed	Low (<5 servings/day)	94.4	92.1	96.2	94.4	91.3	96.6	0.000 (1)	94.5
	Adequate (>5 servings/day)	5.6	3.8	7.9	5.6	3.4	8.7		5.5
Minutes of physical activity	Low (<30)	7.7	5.7	10.0	7.5	5.1	10.6	1.18 (2)	6.1
	Adequate (30–60)	7.0	5.2	9.3	9.0	6.3	12.3		7.0
	High (>60)	85.3	82.3	88.0	83.5	79.3	87.1		86.9
Fasting blood glucose	Normal	79.8	76.3	83.0	83.6	79.2	87.4	1.89 (2)	88.3
	Impaired fasting glycaemia	7.2	5.2	9.6	5.8	3.6	8.8		5.4
	Raised blood glucose	13.1	10.4	16.1	10.6	7.6	14.4		6.2

* The chi-square statistic is significant at the 0.05 level.

**Table 4:** Awareness and health utilization among people in the selected hypertension risk factors.

Variables		Tobacco Use			Alcohol Consumption			BMI		Waist–Hip Ratio		Fasting Blood Glucose		
		Never %	Current %	Former %	Never %	Past %	Current %	Overweight %	Obese %	Overweight %	Obese %	Impaired Fasting Glycaemia %	Raised Blood Glucose %	Overall %
Health worker checked blood pressure	No	47.1	71	57.8	47.5	50.7	61.7	36.8	24.8	48	34.6	46.1	38.1	50.9
	Yes	52.9	29	42.2	52.5	49.3	38.3	63.2	75.2	52	65.4	53.9	61.9	49.1
Health worker diagnosed hypertension	No	78	79.9	71.6	78.8	72.5	77.3	72.7	63.2	76	71.6	72	55.1	77.7
	Yes	22	20.1	28.4	21.2	27.5	22.7	27.3	36.8	24	28.4	28	44.9	22.3
Diagnosed with hypertension in the past year	No	43.4	40.6	52.5	41.9	50.7	46.3	37.3	36.6	40.7	40.3	36.4	34.3	44
	Yes	56.6	59.4	47.5	58.1	49.3	53.8	62.7	63.4	59.3	59.7	63.6	65.7	56
Taken prescribed hypertension medication—past 2 weeks	No	75.7	68.8	77.5	73.6	80.8	77.5	74.5	69.1	79.3	70.6	78.8	60	75.4
	Yes	24.3	31.3	22.5	26.4	19.2	22.5	25.5	30.9	20.7	29.4	21.2	40	24.6
Primary source of healthcare	Self-medicate/alternative therapy	7.6	14.2	17.4	7.6	9.7	13.8	8.6	12.2	11	6.7	9.6	7.5	9.2
	Dispensary/Community Health Worker	34.4	34.7	31.2	35.2	36.3	29.8	30.2	23.3	32.1	34.5	37.4	29	34.2
	Health Center	15.7	16.7	9.6	16.7	10.6	13.9	12.2	11.7	15.1	15.6	14.6	14.3	15.4
	Referral public hospital (former district/provincial)	28.5	25	25.5	27.9	30	26.4	31.8	30.7	27.8	30.2	28.3	33.3	27.9
	Private Healthcare	13.7	9.4	16.2	12.5	13.4	16	17.3	22.1	13.9	13	10	15.9	13.4

**Table 5:** Multiple linear and logistic regressions results.

Variable		SYSTOLIC		DIASTOLIC		HYPERTENSION	
		Coefficients		Coefficients		Odds Ratio	
		(Standard Error)		(Standard Error)		(95% Confidence Interval)	
Tobacco use	Current users	1.46 (1.17)	−3.14 * (1.31)	−0.23 (0.76)	−0.76 (0.78)	0.94 (0.72–1.27)	0.88 (0.61–1.28)
	Former users	7.43 *** (1.52)	−0.79 (1.62)	0.76 (0.90)	−1.15 (0.89)	1.48 ** (1.05–2.10)	0.99 (0.64–1.53)
Age range	30–44		1.93 ** (0.71)		2.04 *** (0.51)		1.33 * (1.00–1.76)
	45–59		11.60 *** (1.23)		6.40 *** (0.68)		3.53 *** (2.6–4.80)
	60–69		22.05 ***(1.85)		6.16 *** (0.98)		5.3 *** (3.70–7.46)
Gender	Males/(females)		5.93 *** (0.88)		0.08 (0.56)		1.02 (0.77–1.35)
Marital Status	Single		1.66 * (0.83)		0.44 (0.58)		0.83 (0.60–1.15)
	Divorced/separated		0.61 (1.16)		0.42 (0.89)		1.07 (0.74–1.55)
	Widowed		0.85 (1.59)		0.17 (0.80)		1.23 (0.87–1.74)
Education Level	Uneducated		0.22 (1.55)		−0.95 (1.05)		0.74 (0.47–1.45)
	Primary		−0.03 (−1.12)		−1.01 (0.81)		0.75 (0.52–1.09)
	Secondary		0.30 (1.06)		0.28 (0.87)		1.0 (0.70–1.43)
Wealth quantile	Second		1.75 (1.06)		−0.07 (0.71)		1.09 (0.80–1.51)
	Middle		1.35 (1.04)		0.30 (0.72)		1.12 (0.80–1.56)
	Fourth		1.06 (1.13)		0.18 80.77)		1.23 (0.87–1.73)
	Fifth (richest)		1.40 (1.34)		0.08 (0.85)		1.0 (0.67–1.50)
Residence	Urban		0.36 (0.91)		0.9 (0.65)		1.11 (0.88–1.39)
Occupational status	Self-employed		1.1 (1.06)		−0.99 (0.82)		0.96 (0.67–1.37)
	Unemployed		0.35 (1.32)		−0.83 (0.98)		0.84 (0.54–1.29)
	Homemaker		1.29 (1.21)		−1.07 (0.89)		0.96 (0.63–1.46)
	Student		3.00 * (1.31)		−0.21 (1.03)		1.5 (0.84–2.76)
	Others (retired, unable to work)		−2.2 (2.89)		−3.41 (1.97)		0.92 (0.50–1.70)
Physical Activity	<30 min		−2.41 (1.53)		−1.17 (1.04)		0.97 (0.68–1.40)
	30–60 min		1.04 (1.31)		1.34 (0.80)		1.17 (0.86–1.60)
Alcohol consumption	Past		3.29 ** (1.14)		0.36 (0.69)		1.18 (0.89–1.57)
	Current		4.08 *** (0.94)		2.73 *** (0.57)		1.68 *** (1.24–2.26)
BMI	Underweight		−6.44 *** (1.14)		−4.04 *** (0.64)		0.55 *** (0.38–0.78)
	Overweight		4.99 *** (0.87)		3.25 *** 80.57)		2.22 ***(1.71–3.56)
	Obese		8.79 *** (1.38)		6.28 *** (0.88)		2.58 *** (1.86–3.56)
Waist–hip ratio	Overweight		2.42 ** (0.76)		1.71 ** (0.51)		1.37 * (1.07–1.74)
	Obese		2.46 ** (0.90)		1.00 (0.62)		1.61 * (1.25–2.09)
	Cons	126.21 (0.52)	113.62 *** (1.57)	82.16 (0.33)	78.29 *** (1.00)	0.23 (0.20–0.26)	0.072 ***(0.04–0.12)
	R^2^		0.2009		0.1294		

* *p* < 0.05; ** *p* < 0.01; *** *p* < 0.001.

**Table 6:** Multiple linear and logit regressions with comparison to all explanatory variables summary statistics.

Variable		SYSTOLIC		DIASTOLIC		HYPERTENSION	
		Coefficients		Coefficients		Odds Ratio	
Tobacco use	Current user	−3.14 *	−3.66 *	−0.756	0.249	0.882	0.873
	Former	−0.79	−0.718	−1.15	−0.849	0.99	0.875
Age range	30–44	1.93 **	1.61 *	2.04 ***	1.64 **	1.33 *	1.32
	45–59	11.6 ***	12.9 ***	6.42 ***	6.3 ***	3.53 ***	3.69 ***
	60–69	22 ***	19.7 ***	6.16 ***	4.29 ***	5.26 ***	4.71 ***
Gender	Males	5.93 ***	5.18 ***	0.0837	0.423	1.02	1.15
Marital status	single	1.66 *	2.07 *	0.438	0.801	0.829	0.967
	divorce/separation	0.606	0.251	0.422	0.105	1.07	1.08
	widowed	0.854	1.57	0.166	0.551	1.23	1.2
Education level	No formal ed.	0.218	0.925	−0.95	0.0787	0.74	0.909
	Primary	−0.0255	1	−1.01	−0.282	0.75	0.937
	Secondary	0.3	0.251	0.278	0.444	1	1.04
Wealth	2 Second	1.75	−0.00978	−0.0652	−0.865	1.1	1.03
	3 Middle	1.35	0.91	0.296	0.476	1.12	1.2
	4 Fourth	1.06	0.492	0.177	0.0618	1.23	1.28
	5 Richest	1.4	0.664	0.0797	−0.09	1	0.979
Residence	Urban	0.364	−0.239	0.902	0.29	1.11	1.04
Occupation	Self-employed	1.07	1.36	−0.986	−0.849	0.962	0.896
	Unemployed	0.353	0.361	−0.834	−0.795	0.836	0.841
	Homemaker	1.29	2.02	−1.07	−0.537	0.956	1.04
	Student	3 *	2.58	−0.212	−0.487	1.52	1.39
	Others	−2.2	−2.12	−3.41	−4.58 *	0.923	0.937
Physical activity	Low	−2.41	−3.76 *	−1.16	−1.2	0.971	0.964
	Recommended	1.04	0.208	1.34	0.601	1.17	1.29
Alcohol	Past	3.29 **	3.38 *	0.36	0.516	1.18	1.22
	Current	4.08 ***	5.08 ***	2.73 ***	2.8 ***	1.68 ***	1.74 **
BMI	Underweight	−6.44 ***	−6.59 ***	−4.04 ***	−3.77 ***	0.546 ***	0.493 **
	Overweight	4.99 ***	4.3 ***	3.25 ***	2.88 ***	2.21 ***	2.02 ***
	Obese	8.79 ***	7.68 ***	6.28 ***	5.82 ***	2.57 ***	2.54 ***
Waist–hip ratio	Overweight	2.42 **		1.71 **	1.54 *	1.37 *	1.41 *
	Obese	2.46 **		1.33 *	1.36	1.61 ***	1.63 **
CVD	yes		−0.0108		−0.0427		1.33
Fruit	Adequate		−1.39		−2.01		1.1
Vegetables	Adequate		−4.53 *		0.405		0.707
Secondhand smoking			−1.67		−1.03		
Cholesterol			1.19 **		0.946 ***		1.2 ***
Waist–hip ratio			12.4 **				
Blood glucose			0.471				
Fasting blood glucose	Impaired glycaemia				0.801		1.19
	Raised blood sugar				2.52 *		2.29 ***
Cons		114 ***	100 ***	78.3 ***	75.5 ***	0.0721 ***	0.0298 ***

* *p* < 0.05; ** *p* < 0.01; *** *p* < 0.001.

## Data Availability

The data presented in this study are available on reasonable request from the corresponding author.
